# Feasibility and Methodological Consideration of Using Wearable Devices to Examine the Relationship Between Sleep and Balance: Case Report With 2 College Students

**DOI:** 10.2196/93019

**Published:** 2026-10-09

**Authors:** Yuan Li, Hongwu Wang, Raghuveer Chandrashekhar, Jordan Major, Mahek Chandna, Stefanie C Bodison

**Affiliations:** 1 Department of Occupational Therapy, University of Florida, 1225 Center Dr, Gainesville, FL, 32610-3007, United States, 1 3522739934

**Keywords:** wearable devices, sleep, balance, longitudinal, case report

## Abstract

This exploratory case study primarily examined the feasibility of using consumer-grade wearable devices for longitudinal sleep tracking and observed descriptive visual trends between sleep patterns and balance performance. Two college students participated over 4 months. Participant 1 used an Apple Watch Series 5 and an OURA ring; participant 2 used a Fitbit Charge 5 and an OURA ring. Data were collected continuously alongside daily sleep diaries and monthly sensory organization test (SOT) balance assessments. As a primary feasibility outcome, missing data rates were initially high (up to 75.2%) but improved significantly after implementing structured weekly check-ins, dropping to 14.1% and 20.3% for the wrist devices, 9.4% and 25% for the OURA rings, and 0% and 6.25% for the sleep diaries in the optimized phase. Wearables showed varying concordance with sleep diaries for time in bed (TIB), with the Apple Watch showing the least bias (–0.6 min) and the Fitbit and OURA overestimating TIB. While devices showed excellent within-participant agreement for total sleep time (intraclass correlation coefficient=0.97‐0.99), agreement was poor for specific sleep stages. Regarding secondary exploratory observations, descriptive visual trends showed that fluctuations in sleep timing and duration coincided with changes in SOT visual subscale scores. In conclusion, consumer wearables are feasible for long-term sleep monitoring when supported by active compliance protocols. While sleep consistency was visually patterned alongside postural stability, these balance findings are strictly exploratory, hypothesis-generating, and not generalizable due to the small sample size and potential practice effects from repeated testing.

## Introduction

Sleep is a critical component of health, influencing physical restoration, cognitive function, and emotional stability [1-3]. Quality sleep is essential for maintaining an active lifestyle. According to the National Sleep Foundation, 35% of US adults sleep less than the recommended 7 hours per night, leading to various health issues [4]. Poor sleep quality is associated with impaired cognitive function, mood disturbances, and an increased risk of chronic diseases [2]. Improving sleep can alleviate pain symptoms and enhance overall well-being [5-7]. College students also experience significant sleep disturbances due to lifestyle factors and academic pressures. Studies found that 70% of college students report insufficient sleep, negatively impacting academic performance and mental health [8,9]. College students are ideal for assessing the feasibility of sleep and balance measurements, given their high acceptance of technology and similar patterns of poor sleep, and they provide valuable knowledge and insights that can be applied to broader populations, including older adults.

Balance is a critical aspect of daily life, and its impairment can lead to falls, especially among vulnerable populations such as older adults and those with certain medical conditions [10]. The relationship between sleep quality and balance is multifaceted. Various studies have shown that sleep deprivation and poor sleep quality negatively affect balance and postural stability. Poor sleep quality has been associated with increased postural sway and a higher risk of falls in the general population [11-13]. Sleep deprivation has been associated with impaired integration of somatosensory, visual, and vestibular contributions to balance and motor coordination [14,15]. Declines in autonomic nervous activity, particularly cardiac vagal modulation, due to decreased nocturnal parasympathetic activity can impair sleep and balance [5]. Increased insomnia severity is associated with a decline in balance, especially among older women, leading to a higher risk of falls [16]. A growing body of research [17-19] further supports the association between sleep disruptions and poorer postural control. However, most prior investigations have relied on cross-sectional designs, limiting the ability to observe longitudinal changes in sleep and balance interactions. Despite the critical interplay between sleep and balance, sleep was not routinely assessed during regular balance assessment, partially due to the lack of a reliable and objective way to longitudinally examine it [19]. Traditional methods of sleep assessment, such as self-reported sleep diaries [20,21] or in-lab polysomnography, present challenges for many individuals due to cognitive decline, memory impairment, mobility issues, and high costs [22]. The advent of wearable technology has revolutionized the way we monitor sleep. Devices such as the Fitbit [23], OURA ring [24], and Apple Watch [25] provide continuous, unobtrusive means to track various sleep parameters. These wearables have gained popularity for their convenience and real-time feedback on sleep patterns. However, the accuracy and reliability of these devices compared to traditional polysomnography and sleep diaries remain areas of ongoing research. A list of review and validation studies [19,24-29] examined the landscape of consumer sleep technologies in sleep tracking, emphasizing the potential and limitations of these devices in accurately capturing sleep data. Fitbit models generally demonstrate moderate-to-high accuracy in total sleep time (TST) estimation but show limitations in detecting wake after sleep onset and sleep stages [28]. The OURA ring has been shown to reliably capture TST but exhibits variable performance for distinguishing specific sleep stages [30]. Apple Watch devices similarly offer good agreement for TST but less consistency for detailed sleep architecture [30]. Prior research found that subjective TST, as measured by a sleep diary, is generally comparable to objective measures among healthy adults [31]. While existing studies often highlight the short-term feasibility and accuracy of wearable devices, the long-term use of these devices for sleep tracking and the longitudinal impact of sleep on health outcomes, such as balance or mobility, remain unclear. These findings underscore the need for feasibility studies assessing not only sleep measurement accuracy but also real-world usability, data completeness, and participant compliance over extended monitoring periods.

This study primarily explores the feasibility of long-term wearable sleep tracking and observes exploratory, descriptive trends regarding its potential visual correspondence with balance performance. We hypothesized that wearable-derived sleep variability would visually align with changes in balance performance over time. By comparing sleep data from different wearables with a traditional sleep diary and exploring visual fluctuations between sleep parameters and balance measures, this research seeks to provide insights into the use of wearables for long-term sleep tracking and health monitoring. Exploratory studies are particularly suited for in-depth methodological exploration, especially when the goal is to identify implementation challenges, user compliance, and data completeness and to refine protocols for future large-scale research [32]. The findings could help develop more effective interventions and recommendations to improve sleep quality and balance, ultimately enhancing overall health and well-being.

## Methods

### Ethical Considerations

This study was conducted from July 2023 to December 2023 and was approved by the University of Florida Institutional Review Board as exempt under Protocol #ET00042583. Participants provided informed consent electronically prior to study enrollment, in accordance with institutional review board exempt protocol guidelines. Data were deidentified to safeguard participant information. Participants were not compensated for their participation. All procedures were performed in accordance with the ethical standards of the institutional and/or national research committee and with the 1964 Helsinki Declaration and its later amendments or comparable ethical standards.

### Study Procedure and Outcome Measures

The study explored the feasibility of long-term wearable sleep tracking and observed exploratory, descriptive trends regarding its potential visual correspondence with balance performance using different wearable technologies (Apple Watch, OURA ring, and Fitbit). Participants were assigned different wearable devices to assess device-specific feasibility and sleep-tracking variability. Two students (1 undergraduate and 1 graduate student) volunteered for this study and were recruited through convenience sampling. The participants were selected based on their willingness to complete a daily sleep diary, regular use of a wearable device, and availability for repeated balance assessments over the study period. Participant 1 wore an Apple Watch Series 5 and an OURA ring (Figure 1A). Participant 2 wore a Fitbit Charge 5 and an OURA ring for four months (Figure 1B). The devices were worn according to the manufacturer’s instructions. Both participants documented time in bed (TIB) and sleep onset time using the National Sleep Foundation Sleep Diary [33]. Both participants were trained to use the diary until they felt confident enough to log their daily sleep. Wearable sleep data were also cross-referenced with self-reported TIB via sleep diary [33]. Sleep diaries are widely recognized as a standard subjective tool for assessing sleep patterns over time. Previous validation studies have shown that self-reported TST from sleep diaries demonstrates reasonable agreement with objective measures such as polysomnography and actigraphy, particularly among healthy adults [4,21]. Balance was assessed monthly with sensory organization tests (SOTs) on the Bertec Balance Advantage system (Figure 2) [34]. Balance assessments were conducted between 9 AM and 11 AM to minimize circadian variability and were standardized across sessions. The participant was instructed to complete up to 18 trials, divided into 3 trials for each of 6 sensory scenarios (Table 1). Balance was assessed monthly (4 times over 4 months). The system generates equilibrium scores to quantify the evaluation sway. Equilibrium scores range from 0% to 100%, with 100% indicating perfect stability and 0% indicating fall [35,36]. A list of sensory scores is calculated by the software, including somatosensory (SOM), visual (VIS), vestibular (VEST), and preference (PREF) scores, as well as composite scores, to provide an understanding of which system is impaired [37]. This case study was reported in accordance with the CARE (Case Report) guidelines (Checklist 1).

**Figure 1.**
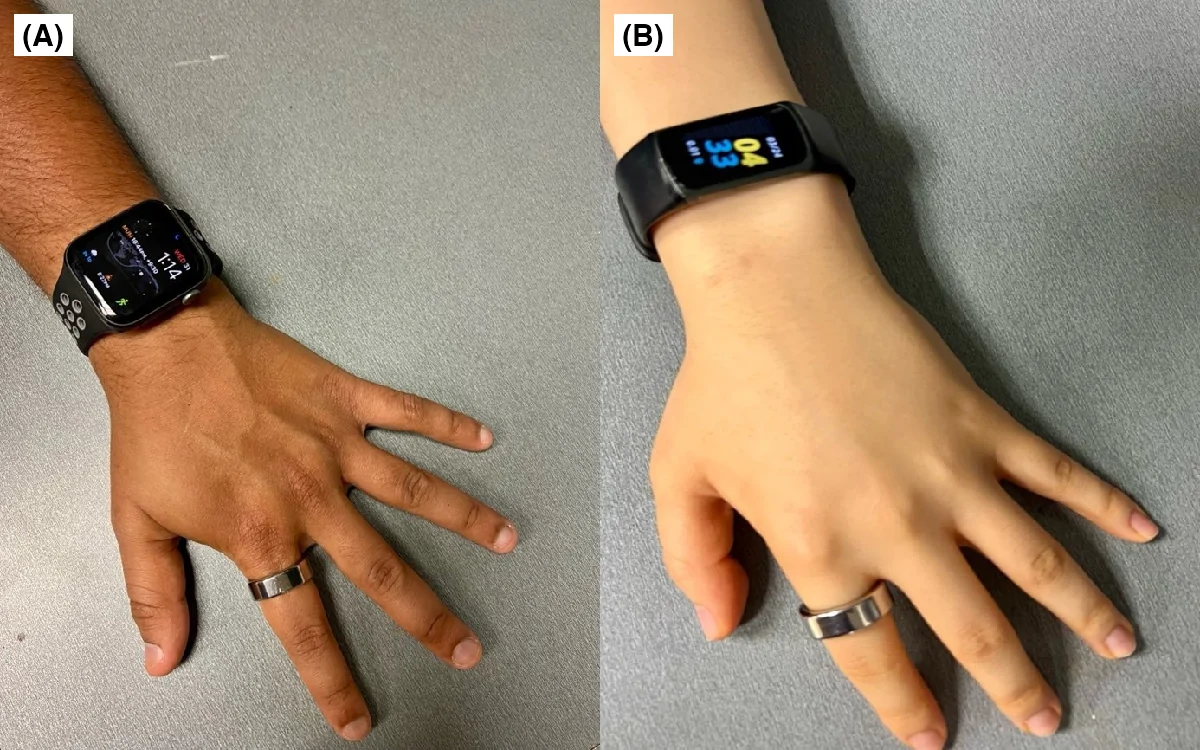
Device placement for long-term sleep monitoring during a 4-month longitudinal feasibility case study (July-December 2023) at the University of Florida. (A) Participant 1 wearing an OURA Ring Gen 3 on the index finger and an Apple Watch Series 5 on the wrist. (B) Participant 2 wearing an OURA Ring Gen 3 on the index finger and a Fitbit Charge 5 on the wrist.

**Figure 2.**
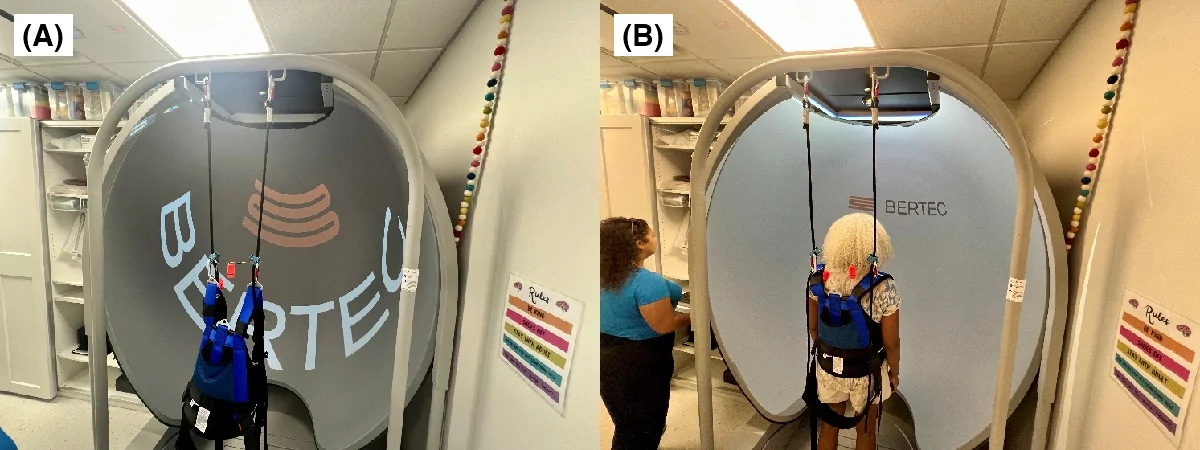
Bertec Balance Advantage system used to conduct monthly sensory organization tests (SOTs) for 2 healthy college students during a 4-month longitudinal feasibility case study (July-December 2023) at the University of Florida. (A) The unoccupied system with the suspended safety harness. (B) A person standing in the system wearing the safety harness for balance assessment.

**Table 1.** Six test conditions of the Bertec Balance Advantage sensory organization test (SOT) used to assess monthly balance in 2 healthy college students during a 4-month longitudinal feasibility case study (July-December 2023) at the University of Florida.

Condition	Vision	VR^a^ environment	Surface
1	Eyes open	Fixed	Fixed
2	Eyes closed	Fixed	Fixed
3	Eyes open	Moving	Fixed
4	Eyes open	Fixed	Moving
5	Eyes closed	Fixed	Moving
6	Eyes open	Moving	Moving

a VR: virtual reality.

### Data Extraction From Wearable Devices and Balance Assessment

The sleep activities and patterns were collected from the Fitbit Charge 5, OURA ring, and Apple Watch Series 5. Participants wore the devices from bedtime until morning, except during showers. We followed the manufacturers’ manuals for data extraction. Fitbit data were downloaded through the Fitbit web interface, OURA data were obtained via the OURA web portal, and Apple Watch data were exported using the “Health Export CSV” app. The extracted sleep variables included daily sleep start time (HH:MM:SS), TIB (minutes), TST (minutes), sleep efficiency (SE, %), wake after sleep onset (WASO, minutes), and different sleep stages duration (light sleep, rapid eye movement [REM] sleep, deep sleep). The Sensory Organization Test data for both participants were downloaded using the Bertec Balance Advantage Dynamic System software, resulting in a printable document for the 4 time points.

### Data Analysis

The collected sleep and balance data were analyzed using descriptive statistical methods and visualizations. Descriptive statistics were used to summarize sleep data collected from wearable devices and the sleep diary over the 4 months to explore agreement between the 2 sources. Parameters such as mean, SD, and range were calculated for each sleep measure. Intraclass correlation coefficients (ICCs) were calculated to assess agreement among wearable devices for various sleep parameters. The ICC values were interpreted as follows: excellent (0.75‐1.00), good (0.60‐0.74), moderate (0.40‐0.59), and poor (<0.40). For all Bland-Altman and ICC analyses, only complete paired observations between each wearable device and the sleep diary were included. Data points with missing values in either the wearable device recording or the sleep diary entry for a given day were excluded from agreement calculations. Following statistical best practices [38], we did not report *P* values and effect sizes. The feasibility of using wearables for long-term sleep monitoring was evaluated by calculating the proportion of missing data for each device and the sleep diary. Mean, SD, and range were calculated for each balance measure. We used data visualization techniques to see the relationships between TST and different sensory scores over time. Balance data visualized in figures were plotted as difference scores (participant’s raw score minus demographic-matched reference values) to visually track deviations from normative stability over time. No inferential statistics were applied due to the exploratory nature of the study. All analyses and data visualization were performed using R Studio 4.2.2 [39].

## Results

### Sleep and Balance Data Descriptive Results

Participant 1 exhibited an average TIB (logged from the diary) of 6.66 (SD 0.48), 6.49 (SD 0.79), 5.92 (SD 1.11), and 5.95 (SD 1.07) hours for the 2 weeks before each balance assessment, respectively. In contrast, participant 2 showed longer average TIB values of 6.94 (SD 2.51), 7.29 (SD 2.24), 7.46 (SD 2.42), and 7.5 (SD 2.25) hours, respectively. The means and SDs of the average TST from wearables for the 2 weeks before each balance assessment date are summarized in Table 2. The 4 monthly balance assessment SOT scores, along with participants’ matched demographic reference scores, are listed in Table 3.

**Table 2.** Summary of average total sleep time and time in bed for the 2 weeks prior to each balance assessment, recorded across a 4-month longitudinal feasibility case study (July-December 2023) for 2 healthy college students at the University of Florida.

ID and date	Two weeks’ mean (SD) of before the balance assessment	
	TIB^a^ (h)	TST^b^ (h)
Participant 1		
July 27, 2023	6.66 (0.48)	5.94 (0.94)
August 25, 2023	6.49 (0.79)	5.94 (0.85)
September 28, 2023	5.92 (1.11)	5.89 (0.91)
October 26, 2023	5.95 (1.07)	5.84 (0.92)
Participant 2		
July 27, 2023	6.94 (2.51)	6.55 (1.97)
August 25, 2023	7.29 (2.24)	6.52 (2.09)
September 28, 2023	7.46 (2.42)	6.51 (2.00)
October 26, 2023	7.5 (2.25)	6.46 (1.87)

a TIB: time in bed.

b TST: total sleep time.

**Table 3.** Monthly sensory organization test (SOT) balance scores alongside demographic-matched reference values for 2 healthy college students evaluated over a 4-month longitudinal feasibility case study (July-December 2023) at the University of Florida.

ID and date	COM^a^ (COM_R), mean (SD)	SOM^b^ (SOM_R), mean (SD)	VIS^c^ (VIS_R), mean (SD)	VEST^d^ (VEST_R), mean (SD)	PREF^e^ (PREF_R), mean (SD)
Participant 1					
July 27, 2023	67 (70)	92 (90)	70 (74)	54 (55)	100 (86)
August 25, 2023	65 (70)	96 (90)	83 (74)	44 (55)	117 (86)
September 28, 2023	67 (70)	107 (90)	80 (74)	72 (55)	94 (86)
October 26, 2023	71 (70)	93 (90)	88 (74)	70 (55)	102 (86)
Participant 2					
July 27, 2023	57 (64)	97 (100)	48 (77)	39 (33)	93 (98)
August 25, 2023	62 (64)	99 (100)	78 (77)	46 (33)	88 (98)
September 28, 2023	66 (64)	100 (100)	75 (77)	65 (33)	84 (98)
October 26, 2023	68 (64)	105 (100)	76 (77)	59 (33)	94 (98)

a COM: center of mass.

b SOM: somatosensory.

c VIS: visual.

d VEST: vestibular.

e PREF: preference.

### Missing Data Rate and Protocol Refinement

Following the protocol refinement on September 15, 2023, we introduced a structured approach involving weekly data checkups and reminders. Prior to this refinement, missing data rates were substantially higher across devices; however, this intervention successfully improved the completeness and reliability of the collected data, as shown in Tables 4 and 5.

**Table 4.** Summary of missing sleep data percentages before and after protocol refinement (September 15, 2023) for consumer wearables and sleep diaries used by participant 1 during a 4-month longitudinal feasibility case study (July-December 2023) at the University of Florida.

Participant 1	OURA	Apple Watch	Sleep diary (%)
Before protocol (September 15)	75.2% missing	36.4% missing	69.4
After protocol (September 15)	9.4% missing	14.1% missing	6.25

**Table 5.** Summary of missing sleep data percentages before and after protocol refinement (September 15, 2023) for consumer wearables and sleep diaries used by participant 2 during a 4-month longitudinal feasibility case study (July-December 2023) at the University of Florida.

Participant 2	OURA (%)	Fitbit (%)	Sleep diary (%)
Before protocol (September 15)	40.3	65.3	36.3
After protocol (September 15)	25	20.3	0

### Within-Participant Agreement Between Wearables and Sleep Diary

The Bland-Altman plots (Figure 3) assessed agreement between wearable-derived TIB and self-reported sleep diary entries. Each plot depicted the mean bias (solid line) and the 95% limits of agreement (LoA, dashed lines) across all paired observations. Only complete, paired daily measurements between each wearable device and the sleep diary were included; incomplete pairs were treated as missing and excluded from the analysis. For participant 1, the Apple Watch showed the highest agreement with the sleep diary, with a minimal mean bias of 0.6 minutes (95% CI –10 to 8.8 min). The 95% LoA indicated that differences between the 2 methods generally ranged from –1.41 to 1.43 hours. The OURA ring slightly overestimated TIB relative to the sleep diary, with a mean bias of 13.8 minutes (95% CI 3-24 min). The 95% LoA ranged from –1.02 to 1.47 hours, indicating moderate variability. For participant 2, the Fitbit Charge 5 overestimated TIB compared to the sleep diary, showing a mean bias of 25.9 minutes. The 95% LoA for these differences ranged from –1.31 to 2.17 hours. The OURA ring also overestimated TIB, revealing a mean bias of 25.5 minutes compared to the sleep diary.

**Figure 3.**
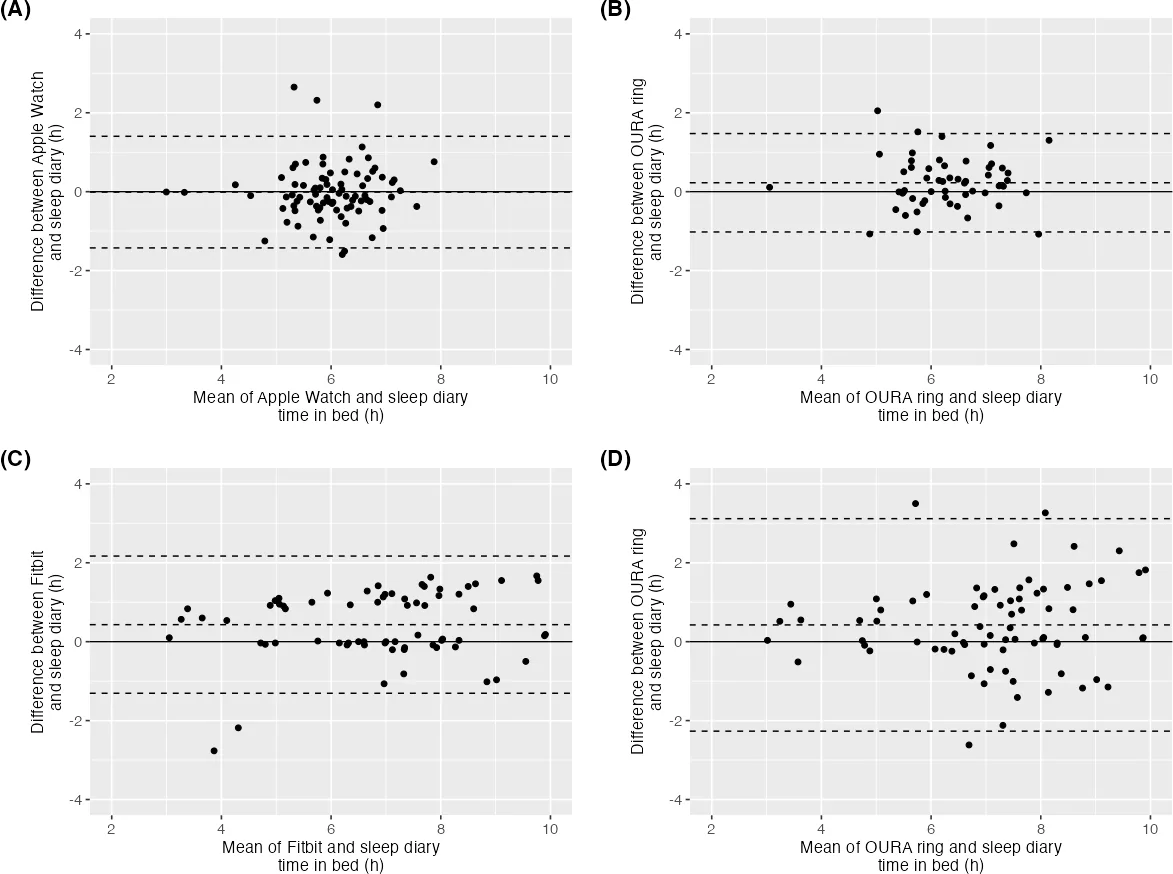
Bland-Altman plots demonstrating within-participant agreement for time in bed (TIB) between consumer wearable devices and self-reported sleep diaries for 2 healthy college students over a 4-month longitudinal feasibility case study (July-December 2023) at the University of Florida. (A) Bland-Altman plot: Apple Watch time in bed and sleep diary. (B) Bland-Altman plot for OURA ring time in bed and sleep diary. (C) Bland-Altman plot: Fitbit time in bed and sleep diary. (D) Bland-Altman plot for OURA ring time in bed and sleep diary.

Overall, the Apple Watch Series 5 showed the strongest agreement with the sleep diary (underestimating TIB by less than 1 minute on average), whereas the Fitbit Charge 5 overestimated TIB by approximately 25.9 minutes. The OURA ring consistently overestimated TIB across both participants (by approximately 13.8 minutes for participant 1 and 25.5 minutes for participant 2). Effect sizes, expressed purely as mean differences and LoA ranges, are emphasized in this interpretation. In accordance with the exploratory nature of this study, no inferential statistics were applied.

### Start Time and TST From Wearables and a Diary

Figure 4 shows the visual trends and fluctuations of participants’ TST and start times. There was a notable increase in variability in both sleep start times and TST over the observed period. For participant 1, late August and late September visually coincided with greater fluctuations in sleep patterns. Over the observed period, participant 2’s sleep start times varied widely, ranging from 10 PM to 2 AM, with most occurring around 1 AM. A slight increase in TST was observed around the second balance assessment.

**Figure 4.**
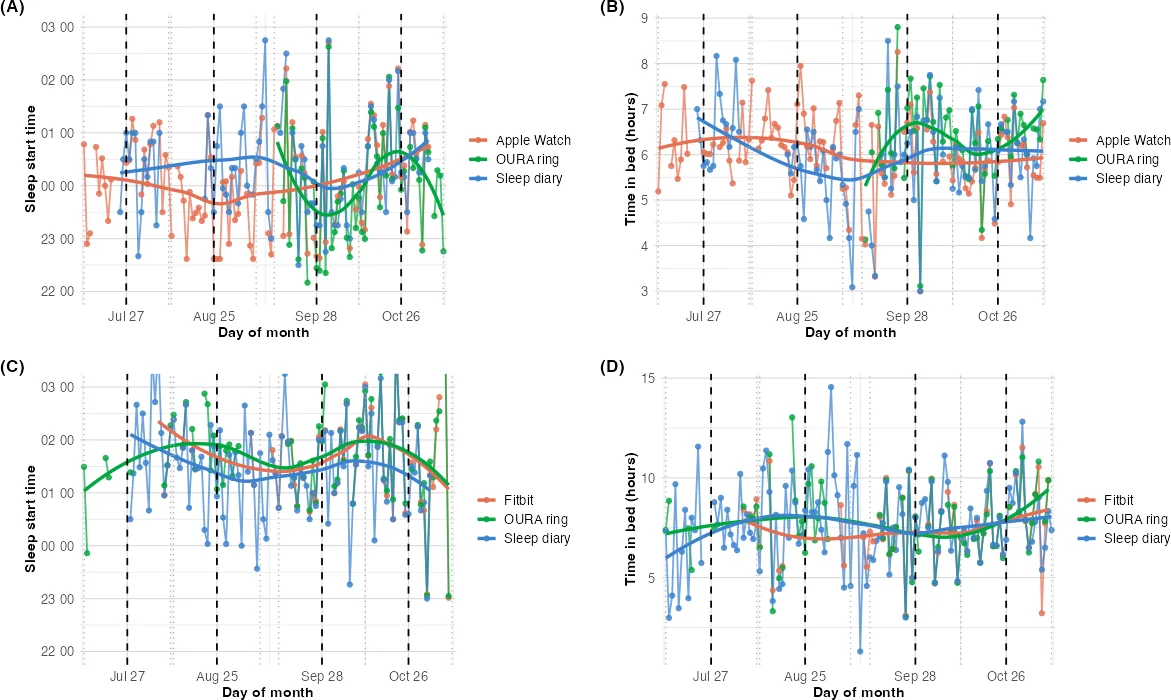
Longitudinal visual trends comparing daily sleep start times and total sleep time (TST) tracked via consumer wearables and self-reported sleep diaries for 2 healthy college students over a 4-month feasibility case study (July-December 2023) at the University of Florida. (A) Case 1 sleep start time. (B) Case 1 time in bed. (C) Case 2 sleep start time. (D) Case 2 time in bed.

### Agreement Between Wearables: ICC Results

Because participant 1 wore the Apple Watch and participant 2 wore the Fitbit Charge 5, these 2 wrist-worn devices cannot be directly compared within the same individual in this study; all ICC and Bland-Altman agreement calculations are strictly between each individual’s specific wearable and their diary. The results showed excellent agreement on TST, with ICC values ranging from 0.97 to 0.99, indicating that all devices provided consistent measurements for TIB and TST. However, the agreement was moderate to good for REM and light sleep and poor for WASO, deep sleep, and SE. For participant 1, comparisons between the OURA ring and Apple Watch demonstrated excellent agreement for TIB (ICC=0.91; 95% CI 0.49‐0.97) and TST (ICC=0.97; 95% CI 0.95‐0.98), good agreement for REM sleep (ICC=0.76; 95% CI 0.58‐0.86), and moderate agreement for light sleep (ICC=0.54; 95% CI –0.19 to 0.81; Table 6). Poor agreement was observed for WASO, deep sleep, and SE (ICCs<0.30).

**Table 6.** Intraclass correlation coefficients (ICCs) assessing device agreement across various sleep parameters between consumer wearables (Apple Watch Series 5, Fitbit Charge 5, OURA Ring Gen 3) worn by 2 healthy college students during a 4-month longitudinal feasibility case study (July-December 2023) at the University of Florida.

Participant and device comparison	TIB^a^, ICC (95% CI)	TST^b^, ICC (95% CI)	WASO^c^, ICC (95% CI)	REM^d^, ICC (95% CI)	Light, ICC (95% CI)	Deep, ICC (95% CI)	SE^e^, ICC (95% CI)
Participant 1: OURA vs Apple Watch	0.91 (0.49 to 0.97); excellent	0.97 (0.95 to 0.98); excellent	0.27 (–0.18 to 0.56); poor	0.76 (0.58 to 0.86); good	0.54 (–0.19 to 0.81); moderate	0.11 (–0.11 to 0.35); poor	0.053 (–0.23 to 0.32); poor
Participant 2: OURA vs Fitbit	0.99 (0.98 to 0.99); excellent	0.99 (0.97 to 1); excellent	0.41 (–0.21 to 0.74); poor	0.59 (–0.08 to 0.82); moderate	0.8 (0.58 to 0.89); excellent	0.66 (0.41 to 0.80); good	0.44 (0.04 to 0.67); good

a TIB: time in bed.

b TST: total sleep time.

c WASO: wake after sleep onset.

d REM: rapid eye movement.

e SE: sleep efficiency.

For participant 2, comparisons between the OURA Ring and Fitbit Charge 5 showed excellent agreement for TIB (ICC=0.99; 95% CI 0.98‐0.99), TST (ICC=0.99; 95% CI 0.97‐1.00), and light sleep (ICC=0.80; 95% CI 0.58‐0.89). Good agreement was observed for deep sleep (ICC=0.66; 95% CI 0.41‐0.80), while moderate-to-poor agreement was found for REM sleep, WASO, and SE. Overall, TIB and TST showed strong agreement across devices, whereas stage-specific metrics, such as deep sleep, REM, and WASO, showed greater variability, reflecting challenges in wearable sleep stage detection.

### Visual Correspondence Between Sleep and Balance

For participant 1 (Figures 5 and 6), there was an exploratory visual trend toward more stable composite balance difference scores from July to October, particularly during periods of earlier sleep start times and increased TIB. This descriptive trend suggests that more consistent sleep patterns visually coincide with more stable somatosensory processing scores. Similarly, the VIS and VEST scores showed gradual stabilization over time. Participant 2 displayed a similar pattern (Figures 7 and 8), visually displaying more consistent balance scores alongside more regular sleep patterns. Visual fluctuations in the VIS score were observed alongside variations in sleep onset times and TST. While all scores remained within typical ranges, these observations suggest that consistent sleep patterns may pattern together with steadier balance performance in these 2 individuals. It is critical to note that the observed gradual improvements in composite and sensory scores over the 4 monthly SOT assessments, particularly for participant 2, may heavily reflect familiarization and practice effects rather than true physiological adaptations to sleep patterns.

**Figure 5.**
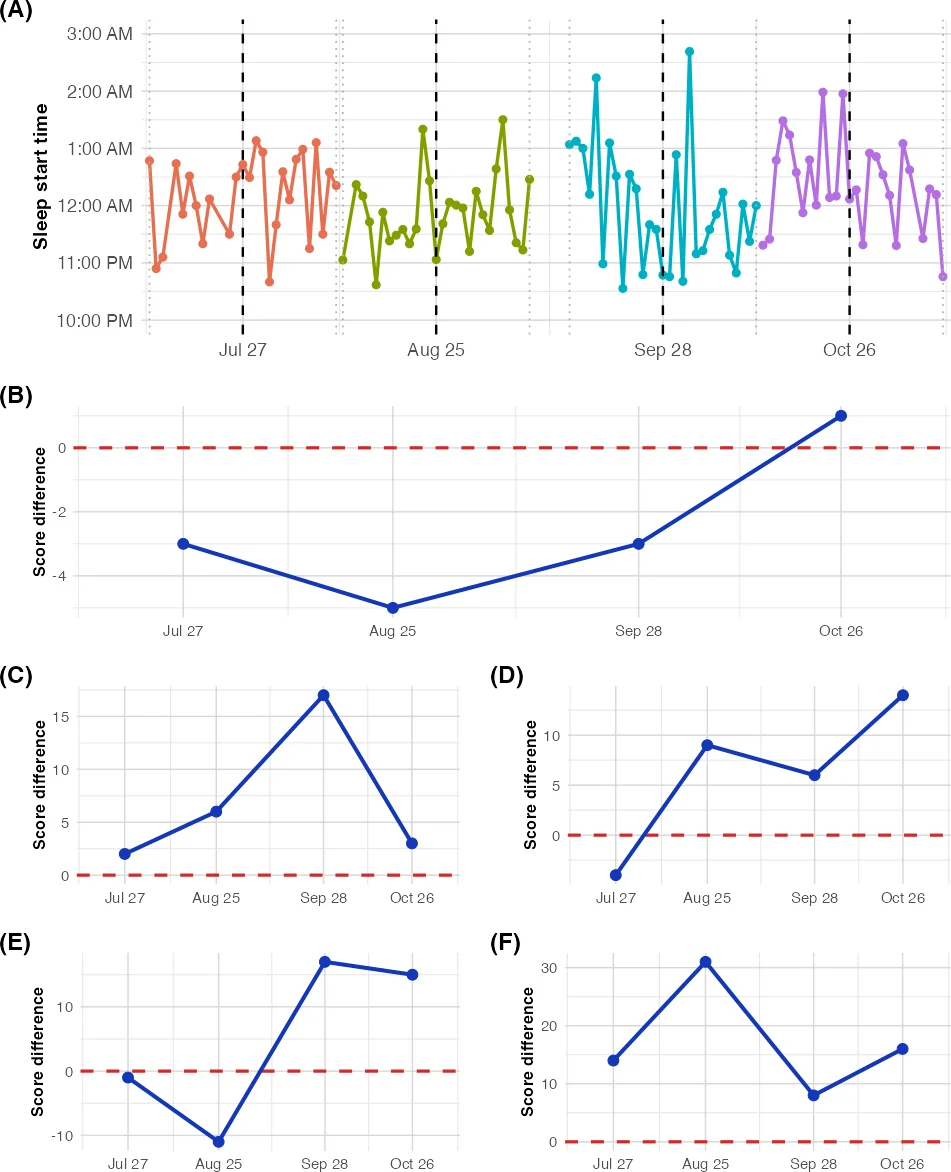
Exploratory visual trends mapping daily sleep onset time against monthly balance score differences (from demographic-matched references) for participant 1 over a 4-month longitudinal feasibility case study (July-December 2023) at the University of Florida. (A) Participant 1 average sleep start time. (B) Composite score difference. (C) SOM score difference. (D) VIS score difference. (E) VEST score difference. (F) PREF score difference. PREF: preference; SOM: somatosensory; VEST: vestibular; VIS: visual.

**Figure 6.**
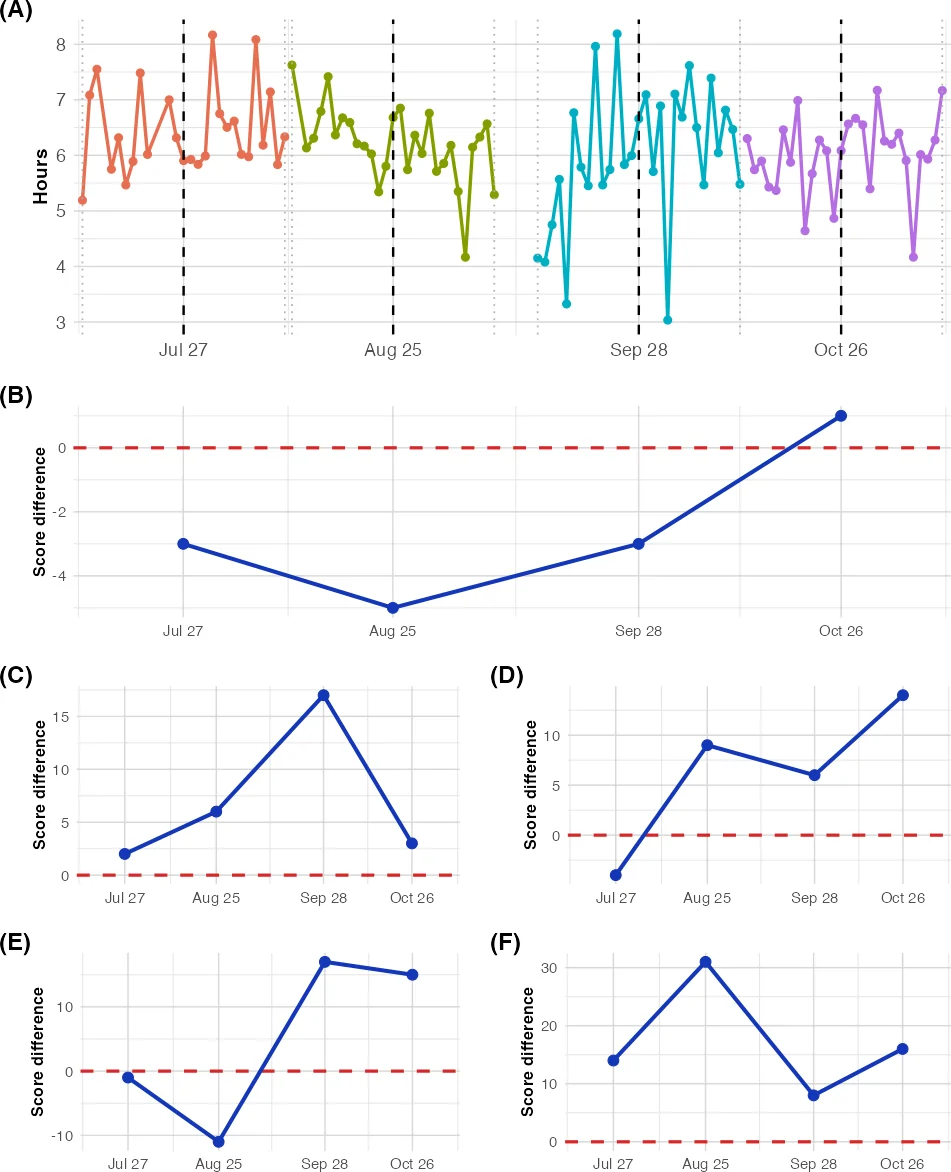
Exploratory visual trends mapping daily time in bed (TIB) against monthly balance score differences (from demographic-matched references) for participant 1 over a 4-month longitudinal feasibility case study (July-December 2023) at the University of Florida. (A) Participant 1 average time in bed. (B) Composite score difference. (C) SOM score difference. (D) VIS score difference. (E) VEST score difference. (F) PREF score difference. PREF: preference; SOM: somatosensory; VEST: vestibular; VIS: visual.

**Figure 7.**
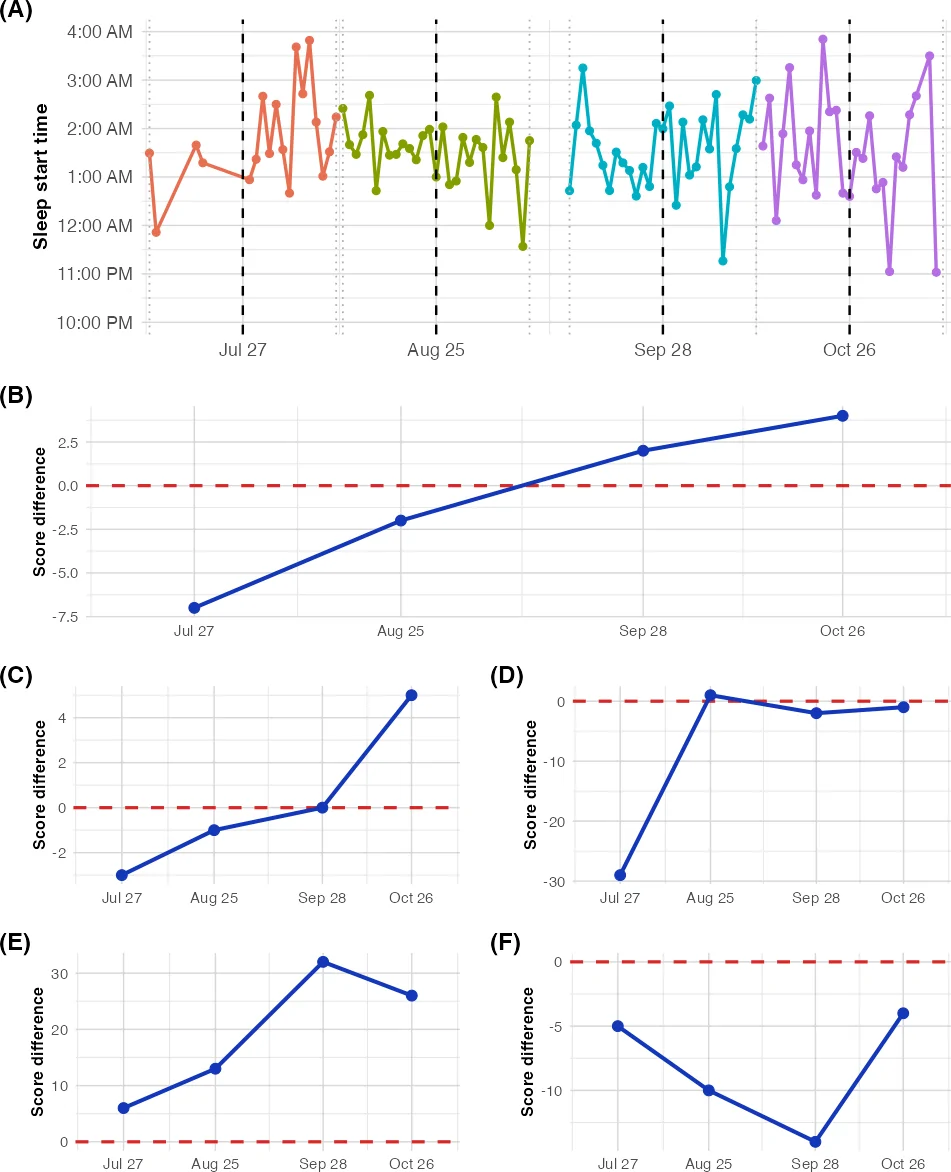
Exploratory visual trends mapping daily sleep onset time against monthly balance score differences (from demographic-matched references) for participant 2 over a 4-month longitudinal feasibility case study (July-December 2023) at the University of Florida. (A) Participant 2 average sleep start time. (B) Composite score difference. (C) SOM score difference. (D) VIS score difference. (E) VEST score difference. (F) PREF score difference. PREF: preference; SOM: somatosensory; VEST: vestibular; VIS: visual.

**Figure 8.**
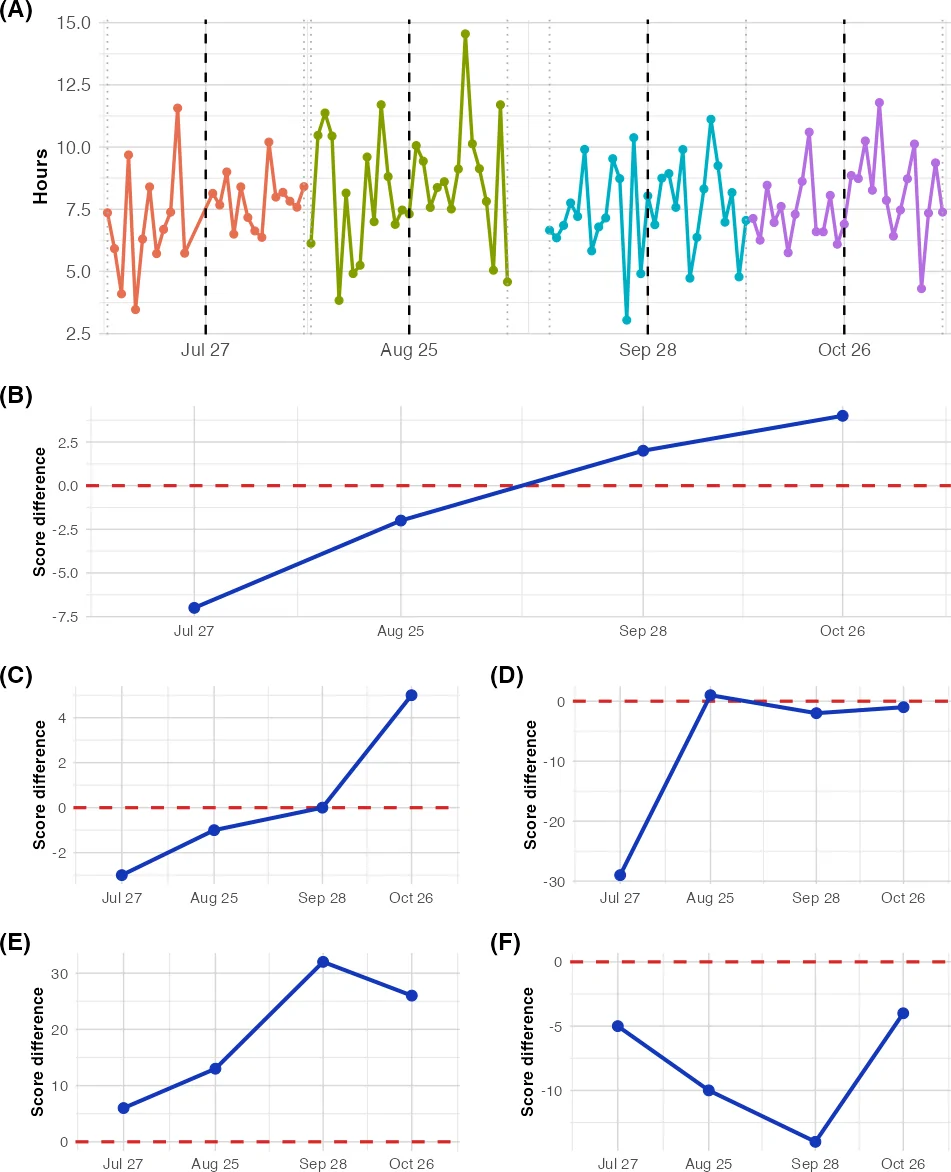
Exploratory visual trends mapping daily time in bed (TIB) against monthly balance score differences (from demographic-matched references) for participant 2 over a 4-month longitudinal feasibility case study (July-December 2023) at the University of Florida. (A) Participant 2 average time in bed. (B) Composite score difference. (C) SOM score difference. (D) VIS score difference. (E) VEST score difference. (F) PREF score difference. PREF: preference; SOM: somatosensory; VEST: vestibular; VIS: visual.

## Discussion

### Brief Summary of Findings

The primary objective of this study was to explore the feasibility of long-term wearable sleep tracking and observe descriptive trends regarding its potential visual correspondence with balance performance. By comparing sleep data from different wearables to a sleep diary and visually exploring fluctuations between sleep parameters and balance measures, this research provided insights into the application of wearables for long-term sleep tracking and health monitoring. Specifically, our findings demonstrated that longitudinal wearable compliance is feasible when supported by structured weekly check-ins and that consumer devices show excellent agreement with one another for TST but vary in their concordance with self-reported TIB. Additionally, exploratory visual analyses suggested that variations in sleep consistency might coincide with fluctuations in postural stability over time. We caution against interpreting these observed trends as causal or generalizable and instead propose this as an illustrative example of how wearables may be used to explore sleep-balance interactions in longitudinal, real-world settings.

### Detailed Discussion of Findings

#### Comparison of Sleep Patterns

Participant 1 and participant 2 demonstrated distinct sleep patterns, with participant 2 exhibiting longer average TIB and greater variability than participant 1. These differences were consistently reflected in both the sleep diary and wearable measurements. Participant 2’s higher variability in TIB aligns with the findings from previous studies indicating that young adults often experience inconsistent sleep schedules due to lifestyle factors [8,40]. These variations in TST and sleep start time also align with findings in the literature that suggest college students often experience irregular sleep schedules due to academic and social commitments [8].

#### Feasibility and Study Compliance

Following protocol refinement midway through the study (September 15, 2023), we introduced a structured approach with weekly data check-ins, reviews, and reminders. Prior to this intervention, missing data rates were high, largely due to common operational challenges, including device charging burdens, data synchronization failures, and the daily adherence required for the sleep diary. This restructured approach demonstrated the effectiveness of regular monitoring and reminders in enhancing data reliability and completeness. Our participant data showed improved compliance with data completion, with reduced missing data (eg, OURA ring missingness dropping from 75.2% to 9.4% for participant 1) and increased wearing time. While our assessment of feasibility was primarily based on data missingness and long-term wear compliance rather than on formal questionnaires on usability, preference, or comfort, these findings emphasize the practical feasibility of using wearables for extended sleep monitoring. Crucially, they highlight that deploying consumer wearables in longitudinal research requires active, ongoing participant engagement to overcome technological and adherence barriers, while acknowledging their limitations in measuring specific sleep parameters among college students.

#### Agreement Between Wearables and Sleep Diaries

The Bland-Altman analyses revealed a small bias in TIB between wearables and sleep diaries. The Apple Watch showed the least bias, underestimating TIB by less than 1 minute compared to the diary, whereas the Fitbit Charge 5 and OURA ring overestimated TIB by approximately 26 minutes and 14 to 25.5 minutes, respectively. This finding is consistent with the literature, suggesting that wearable devices such as Fitbit can slightly overestimate TST due to difficulties in distinguishing between wakefulness and light sleep [17,41,42]. Our study confirms previous findings that wearables generally provide reliable estimates of TST but can vary in their relative agreement across devices. It is important to note that a sleep diary is a subjective measure rather than a clinical gold standard; thus, these comparisons evaluate concordance rather than criterion validity. The excellent agreement in TIB and TST (ICC=0.97‐0.99) between wearables supports their use for monitoring general sleep patterns over extended periods. However, moderate-to-poor agreement in REM, light sleep, and WASO suggests caution when interpreting detailed sleep stage data across different wearables.

#### Balance Performance and Sleep

For participant 1, there is notable variability in sleep start times, which visually coincided with fluctuations in balance composite scores. Specifically, later sleep start times appeared to be visually coincided with greater deviations in balance scores from the reference value. This suggests that both the timing and duration of sleep could potentially be linked to balance performance in an exploratory context. Consistency in sleep start times and sufficient TIB may visually track with more stable balance scores, whereas disruptions, such as irregular start times or insufficient TST, may coincide with deviations in balance due to fatigue or inadequate rest. In contrast, participant 2 shows a consistent upward trend in composite score differences over time, indicating potential improvement in balance throughout the study period. Changes in composite score differences for participant 2 are more gradual yet consistent, suggesting a steadier adaptation in balance assessment, which may also be heavily influenced by a learning or practice effect from repeated Bertec SOT testing.

Overall, both participants demonstrate a potential visual link between sleep characteristics and balance performance. Variability in sleep timing and duration may be visually coincided with balance changes, as indicated by differences in the composite score. However, the degree and pattern of these observed trends differ between the 2 participants, suggesting that individual differences and practice effects may influence how sleep impacts balance. These findings emphasize the methodological importance of regular sleep monitoring for longitudinal assessment of balance performance and highlight the potential role of personalized sleep interventions in future large-scale balance-related health strategies.

#### Different Sensory Domains

##### Somatosensory

The SOM domain evaluates tactile and proprioceptive information from the feet and legs to maintain stability on different surfaces. For participant 1, the SOM score difference initially rises, peaking around the third assessment, indicating a temporary positive change in balance performance before returning to baseline at the fourth assessment. In contrast, participant 2 showed a gradual and consistent improvement over time without returning to baseline. Visual trends from both participants suggest that variability in sleep patterns, including start timing and duration, might be visually coincided with changes in balance performance. Increased variability appeared to coincide with worsening SOM scores. Inconsistent sleep start times in both participants may correlate with deviations in balance performance, potentially due to disrupted circadian rhythms affecting physical stability. This observational trend aligns with existing literature suggesting that sleep quality impacts somatosensory processing since the consolidation of sensorimotor memories enhances somatosensory function [43].

##### Visual

The VIS domain, which measures reliance on visual information to maintain balance, was explored in relation to sleep patterns. Participant 1’s VIS score initially increases, peaking on the second assessment, then drops slightly before increasing again by the fourth assessment. This suggests fluctuations in visual stability, possibly linked to variations in sleep patterns. Participant 2 starts below the reference line and remains stable from the second to the fourth measurement, indicating consistent visual stability over time. These observations align with research indicating that sleep deprivation can impair visual processing and perceptual tasks. Our exploratory findings extend this understanding by suggesting that even minor alterations in sleep timing and duration might visually correspond with changes in visual sensory integration, impacting balance performance.

##### Vestibular

The VEST domain, which assesses the ability to use vestibular cues when both visual and somatosensory information are compromised, was also visually mapped against sleep patterns [44]. For participant 1, the vestibular stability score difference shows a decline between the first and second assessments, followed by an increase by the third assessment. This pattern indicates a temporary decrease in vestibular stability, which may be linked to fluctuations in sleep start times and duration. Specifically, irregular sleep schedules and shorter TST visually correlated with decreased vestibular stability, suggesting that disrupted sleep patterns might potentially relate to vestibular function. Participant 2, on the other hand, demonstrated a steady increase in vestibular stability across the first 2 assessments, indicating continuous improvement, even as sleep start times and durations became more consistent. However, there was a slight decline in the last assessment, which might correspond to renewed variability in sleep patterns. This observation highlighted the potential importance of consistent sleep onset times and adequate TST for maintaining vestibular stability. The gradual improvement in vestibular scores (though potentially confounded by learning effects) with more consistent sleep patterns supports existing literature linking vestibular function to sleep quality. Research indicated [45] that sleep disturbances can exacerbate vestibular symptoms and lead to balance issues. Our findings suggest that consistent sleep start times and sufficient TST might benefit vestibular processing, potentially mitigating balance impairments.

##### Preference

The observed PREF score differences indicate that the ability to rely on various sensory inputs for balance might be influenced by sleep quality. For participant 1, the initial increase in preference score difference from the first two assessments suggested a deviation from the reference value. This indicated a temporary shift in the participant’s reliance on sensory inputs for balance. By September 28, the score decreased, indicating a return to the reference value. Subsequently, a slight increase was observed during the fourth visit. These fluctuations might be attributed to variations in sleep onset and duration, as inconsistent sleep patterns can disrupt the integration of sensory inputs essential for balance. This finding aligns with research demonstrating that sleep supports the flexible use of sensory information, which is crucial for maintaining balance in dynamic environments or when sensory information is unreliable [43]. While our study provides an exploratory visual observation that aligns with theories suggesting better sleep enhances sensory preference adaptability, the exact mechanisms require further investigation. Further research is needed to examine how specific aspects of sleep, such as consistency and duration, influence sensory integration and balance preference.

A key strength of this exploratory study is its longitudinal design, which allows for the observation of intra-individual trends in balance performance over time. By examining individual cases in detail, this study offers a methodological framework for how sleep might influence balance, contributing to the growing body of literature on sensory processing and motor control. Furthermore, the use of consumer wearable devices to collect sleep data enables continuous, real-world monitoring. This approach enhances the ecological validity of the findings and demonstrates a practical method for tracking sleep outside of a clinical laboratory. Additionally, mapping these wearable data against specific sensory domains, such as somatosensory, visual, vestibular, and preference, provides a comprehensive assessment strategy that future, adequately powered studies can use to explore targeted interventions.

### Limitations

Despite its strengths, the study has several limitations that must be acknowledged. The most notable limitation is the small sample size, which includes only 2 participants. This limits the generalizability of the findings, as individual differences may impact the results. Future studies should include a more extensive and diverse sample, accounting for various demographic factors such as age, gender, and health status, to allow a more comprehensive understanding.

Another limitation is the potential learning effect associated with repeated use of the Bertec system. Participants may improve performance over time due to familiarity with the testing procedures rather than actual balance changes [36,46,47]. In a study of this size, these practice effects could potentially explain the observed longitudinal improvements in balance as much as, or more than, sleep-related changes. This could confound results, making it challenging to isolate the impact of sleep on balance. Although adjustment for learning effects was not feasible in this study design due to the small sample size, future studies should consider implementing washout periods, randomizing testing conditions, or using statistical methods to account for practice effects when conducting repeated balance assessments.

Additionally, several important potential confounders, including daily physical activity levels, caffeine and alcohol consumption, psychological stress, and screen use, were not systematically recorded [11,48-50]. These factors are known to influence both sleep quality and balance, and their omission limits the ability to isolate sleep as an independent contributor to balance variability. Future studies should incorporate concurrent monitoring of these variables to more precisely characterize the complex interactions between sleep and postural control.

While sleep diaries offer practical advantages for longitudinal monitoring, they are subject to recall bias and may not capture brief nighttime awakenings as accurately as objective measures. Furthermore, it is important to note that a sleep diary is a subjective tool and not a clinical gold standard for objective sleep architecture, which limits our ability to make definitive criterion validity claims regarding the wearable devices.

Regarding operational feasibility, the missing data before the protocol refinement on September 15, 2023, posed a challenge to the study’s completeness. However, introducing structured weekly data checkups and reminders reduced missing data, thereby improving the study’s reliability. Nevertheless, formal assessments of device comfort, usability, participant preferences, charging burden, and data synchronization issues were not systematically collected, which limits the ability to evaluate overall real-world feasibility.

Despite these limitations, the study offers a methodological foundation for further research on the role of sleep and sensory integration in balance performance. By highlighting descriptive visual trends regarding the potential impact of sleep patterns on balance, this study underscores the importance of considering sleep as a factor in future large-scale postural control research, particularly in populations at risk for sleep disturbances and balance impairments.

### Conclusions

This study demonstrates the feasibility of using consumer-grade wearable devices to monitor sleep and explore its potential visual correspondence with balance performance in a real-world, longitudinal context. The excellent agreement among wearables on key sleep parameters supports their use in longitudinal studies, while observed descriptive trends between sleep consistency and postural stability suggest potential relationships warranting further exploration. These findings have important methodological implications for rehabilitation professionals; for instance, literature suggests that while occupational therapists recognize sleep as an important domain [51], they often lack accessible tools to continuously assess it. Technological advancements in wearable devices offer practical opportunities for therapists to conduct ongoing assessments and tailor personalized care. By leveraging continuous tracking, health care providers can better observe the complex interactions between sleep and mobility over time. Future research must use significantly larger, more diverse sample sizes and account for learning effects from repeated balance tests to validate these exploratory trends and fully evaluate sleep’s role in postural control.

## Supplementary material

10.2196/93019 Checklist 1 CARE checklist.

## Abbreviations

CARE: Case Report
ICC: intraclass correlation coefficient
LoA: limits of agreement
PREF: preference
REM: rapid eye movement
SE: sleep efficiency
SOM: somatosensory
SOT: sensory organization test
TIB: time in bed
TST: total sleep time
VEST: vestibular
VIS: visual
WASO: wake after sleep onset
