# Supplementary material for: Feasibility and Methodological Consideration of Using Wearable Devices to Examine the Relationship Between Sleep and Balance: Case Report With 2 College Students
Source: JMIR Form Res. 2026 Oct 9;10:e93019. doi: 10.2196/93019 (PMC13652396; doi:10.2196/93019)
Supplement: Supplementary file 1 [file formative-v10-e93019-s001.pdf]

CARE
case report guidelines

CARE Checklist of information to include when writing a case report

(cc) BY-NC-ND

Topic	Item	Checklist item description	Reported on Line
Title	1	The diagnosis or intervention of primary focus followed by the words "case report"	Title
Key Words	2	2 to 5 key words that identify diagnoses or interventions in this case report, including "case report"	10
Abstract (no references)	3a	Introduction: What is unique about this case and what does it add to the scientific literature?	13-15
	3b	Main symptoms and/or important clinical findings	21-28
	3c	The main diagnoses, therapeutic interventions, and outcomes	15-21
	3d	Conclusion—What is the main "take-away" lesson(s) from this case?	33-38
Introduction	4	One or two paragraphs summarizing why this case is unique (**may include references**)	40-85
Patient Information	5a	De-identified patient specific information.	110-114
	5b	Primary concerns and symptoms of the patient.	N/A (Healthy participants)
	5c	Medical, family, and psycho-social history including relevant genetic information	N/A (Healthy participants)
	5d	Relevant past interventions with outcomes	N/A
Clinical Findings	6	Describe significant physical examination (PE) and important clinical findings.	Table 3
Timeline	7	Historical and current information from this episode of care organized as a timeline	Tables 2 & 3
Diagnostic Assessment	8a	Diagnostic testing (such as PE, laboratory testing, imaging, surveys).	105-131
	8b	Diagnostic challenges (such as access to testing, financial, or cultural)	133-142
	8c	Diagnosis (including other diagnoses considered)	N/A
	8d	Prognosis (such as staging in oncology) where applicable	N/A
Therapeutic Intervention	9a	Types of therapeutic intervention (such as pharmacologic, surgical, preventive, self-care)	N/A (observational)
	9b	Administration of therapeutic intervention (such as dosage, strength, duration)	N/A
	9c	Changes in therapeutic intervention (with rationale)	N/A
Follow-up and Outcomes	10a	Clinician and patient-assessed outcomes (if available)	144-152
	10b	Important follow-up diagnostic and other test results	N/A
	10c	Intervention adherence and tolerability (How was this assessed?)	225-229 & Table 5
	10d	Adverse and unanticipated events	N/A
Discussion	11a	A scientific discussion of the strengths AND limitations associated with this case report	359-405
	11b	Discussion of the relevant medical literature **with references**.	245-343
	11c	The scientific rationale for any conclusions (including assessment of possible causes)	232-243
	11d	The primary "take-away" lessons of this case report (without references) in a one paragraph conclusion	407-409
Patient Perspective	12	The patient should share their perspective in one to two paragraphs on the treatment(s) they received	Not Reported
Informed Consent	13	Did the patient give informed consent? Please provide if requested	Yes ☑ No ☐
